# COVID‐19 in Western Australia: ‘The last straw’ and hopes for a ‘new normal’ for parents of children with long‐term conditions

**DOI:** 10.1111/hex.13792

**Published:** 2023-06-12

**Authors:** Stephanie Smith, Mary Tallon, James Smith, Lauren Jones, Evalotte Mörelius

**Affiliations:** ^1^ School of Nursing and Midwifery Edith Cowan University Perth Western Australia Australia; ^2^ Curtin Medical School Curtin University Perth Western Australia Australia; ^3^ Telethon Kids Institute Perth Western Australia Australia; ^4^ School of Nursing Curtin University Perth Western Australia Australia; ^5^ Child and Adolescent Health Service Perth Western Australia Australia; ^6^ School of Population Health Curtin University Perth Western Australia Australia; ^7^ School of Medical and Health Sciences Edith Cowan University Perth Western Australia Australia; ^8^ Parent Representative Bunbury Western Australia Australia; ^9^ Department of Health, Medicine and Caring Sciences Linköping University Linköping Sweden

**Keywords:** children, chronic, lived experiences, parents' experiences, qualitative, stress, stress coping

## Abstract

**Background:**

Children with long‐term conditions are vulnerable due to the treatments required for their conditions. Since the start of the coronavirus disease 2019 (COVID‐19) pandemic, Western Australians experienced restrictions that changed daily life activities but were able to return to some of their previous routines due to the restrictions.

**Aim:**

The study explored the stress experiences of parents caring for children with long‐term conditions during COVID‐19 in Western Australia.

**Design and Participants:**

The study was codesigned with a parent representative caring for children with long‐term conditions to ensure essential questions were targeted. Twelve parents of children with various long‐term conditions were recruited. Ten parents completed the qualitative proforma, and two parents were interviewed in November 2020. Interviews were audio‐recorded and transcribed verbatim. Data were anonymised and analysed using reflexive thematic analysis.

**Findings:**

Two themes were produced: (1) ‘Keep my child safe’ describes the children's vulnerabilities due to their long‐term conditions, the adjustments parents' made to keep their children safe and the various consequences faced. (2) ‘COVID‐19's silver lining’ covers the positives of the COVID‐19 pandemic, including their children having fewer infections, the availability of telehealth appointments, relationship improvements and the parent's hopes for a new normal where behaviours prevent transmission of infectious (e.g., hand sanitising).

**Conclusion:**

Western Australia provided a unique context for the COVID‐19 pandemic due to no transmission of the virus severe acute respiratory syndrome coronavirus 2 at the time of the study. The tend and befriend theory aids in explaining the parents' stress experiences, and the application highlights a unique aspect of this theory. Parents tended to their children during COVID‐19, but many could no longer rely on others for connection, support and respite, and became further isolated in attempting to protect their children due to COVID‐19 consequences. The findings highlight that some parents of children with long‐term conditions need specific attention during times of pandemics. Further review is recommended to support parents through the impact of COVID‐19 and similar crises.

**Patient or Public Contribution:**

This study was codesigned with an experienced parent representative who was part of the research team and involved throughout the research process to ensure meaningful end‐user engagement and ensure essential questions and priorities were addressed.

## 1 INTRODUCTION

The coronavirus disease 2019 (COVID‐19) emerged in December 2019 and was declared by the World Health Organisation as a pandemic on 11 March 2020.^1^ COVID‐19 is caused by severe acute respiratory syndrome coronavirus 2 and can result in respiratory distress.^2^

Western Australia (WA) is Australia's largest state, with 33% of the total land mass.^3^ Approximately 610,000 children and young people, of which 40,000 are Aboriginal, live in WA and make up 23% of the state's population.^4^ It is estimated that around 262,300 children aged 0–14 have one chronic condition, and 122,000 have two or more chronic conditions in WA.^4^, ^5^ The first WA case of COVID‐19 was recorded on 21 February 2020,^6^ and the first death on 1 March 2020.^7^ On 15 March 2020, WA was declared a state of emergency.^8^ Gyms and indoor sporting facilities, playgrounds, skate parks and outside gyms in public places were closed.^8^ Australians were told to stay home unless shopping for food and necessities or to address health and medical needs.^8^ From 26 March to 9 April 2020, families were encouraged to keep children home.^8^ One of the government's critical strategies in minimising the impact of COVID‐19 was to close the national and WA state borders to travellers in March 2020.^9^ Other measures included lockdowns, isolation, social distancing^8^ and masks.^10^ WA enforced the strongest COVID‐19 border controls in Australia.^9^ In May 2020, all school students were required to return to school, and restrictions started to ease.^9^

COVID‐19 has significantly affected how everyone lives.^11^ Previous research has confirmed that since the COVID‐19 crisis, parents have experienced stress regarding social distancing, remote learning, financial difficulties and space for themselves.^12^ Yet, there is a lack of literature on parents' experiences caring for children with long‐term conditions, which is already known to cause stress.^13^, ^14^

Long‐term or chronic conditions among children are rising^15^ and refer to a wide range of conditions, illnesses and diseases that tend to be long‐lasting with persistent effects.^16^ Children with long‐term conditions are a vulnerable population dependent on health and education services that have been impacted by the pandemic.^17^ Their parents already face higher mental health burdens as well as higher rates of work loss and financial strain due to COVID‐19.^13^, ^18^ These families and children have been affected as most services required often cannot be delivered outside of a specialist setting, and it is difficult for parents to replace the support their children usually receive.^19^ Families with a child with disabilities are already marginalised. Therefore, consideration of the study context is essential.^20^ For example, it has been stated that the pandemic has further challenged the already difficult situations experienced by parents and their children.^18^

Stressful events may cause discomfort or trigger a stress response, but may also promote family strength and resilience.^21^, ^22^ COVID‐19‐related restrictions have been considered as potentially enhancing stressful events.^22^ Many families have had to learn new ways of adapting to further isolation and profound unpredictability. While many will adapt and grow in resilience, others may experience stress‐related disorders that are previously unknown.^23^ Over time, an accumulation of financial loss, poor sleep, social isolation and unresolved fear may overload the neurobiological pathways that help people adapt to stress.^24^, ^25^ As a result of this overload, the anticipated mental health burden due to COVID‐19 is vast, described as a new type of mass trauma with unprecedented public exposure.^23^

Research is therefore needed to gain insight into the impact of COVID‐19 on the lives of parents caring for children with long‐term conditions and their needs. WA provided a unique context to investigate the effects of COVID‐19 as there was no sustained community transmission at the time of this study, but the threat had been experienced.

This study was conducted in November 2020, just before the controlled interstate border was introduced, allowing very low‐risk states and territories in Australia to travel to WA.^26^ WA, at this time was operating at some level of normality with no community spread. Therefore, this study aimed to explore the experiences and needs of parents caring for children with long‐term conditions concerning the COVID‐19 pandemic and provide recommendations to improve services in preparation for possible future pandemics and crises.

## 2 METHODS

### 2.1 Design

This study used a rapid qualitative approach.^27^, ^28^, ^29^ The study was codesigned with L. J., a parent representative who is a parent caring for children with long‐term conditions. Codesign in this research study included L. J. being part of the research team and involved throughout the research process to ensure meaningful end‐user engagement^30^ and essential questions and priorities were addressed for this parent group. The importance of collaborating with parents caring for children with long‐term conditions has been described previously.^13^, ^31^ L. J. did not participate in the study.

### 2.2 Participants

A purposive sample was recruited from an existing study on parents' experiences of stress caring for a child with chronic conditions,^13^ conducted before COVID‐19 was known and a prominent threat in WA. Parents had been previously recruited via a recognised family support organisation they were registered with.^13^ Inclusion criteria were the parents' children: (a) had at least one long‐term condition, (b) were aged 0–19 years, (c) diagnosed/started treatment within the last 5 years and (d) 6 months postdiagnosis/treatment. Parents were contacted via email and offered the option to complete the open‐ended proforma attached to the email, or if they would prefer to be contacted by telephone and be asked the same questions by a researcher (Box 1). If no response from the email was received within 1 week, a follow‐up telephone call and email were made. If no response was received from the follow‐up, it was assumed that it was not a possibility for the parent to take part. An email template and telephone interview script were used to ensure the participants were provided with standardised guidelines.

Box 1 **Proforma/interview questions**

**Table:** 

Please tell us about your experience of caring for a child with chronic conditions during the coronavirus disease 2019 (COVID‐19) pandemic (e.g., negative or positive impacts to you, your child and family such as access to services/support improvements/barriers). If your situation is different now compared to before the pandemic—please tell us how it is different (if you like, please provide examples). Please use this space to tell us anything you'd like to add (e.g., recommendations of improvements to services experienced during COVID‐19).

Twenty eligible parents were invited to participate; one declined and seven did not respond. A final 12 participants took part in the study. A ‘parent’ was defined in this study as a person with the care responsibilities of the child.

The parents (seven mothers and five fathers) were aged between 31 and 63. One parent identified as Aboriginal Australian, nine as White Australian and two as White British. Two parents were a married couple, and three parents lived in regional WA. Three parents worked full‐time, three worked part‐time and six were full‐time carers. Four parents had more than one child with long‐term conditions.

All names have been changed and do not link to the previous article's pseudonyms^13^ to further protect the parent's anonymity. The children's conditions are also categorised for anonymity purposes. A range of diagnoses was provided; most included more than one health diagnosis and required specialist care from two or more specialist health teams. Table 1 outlines the participants' profiles.

**Table 1:** Participant profiles.

	Parent	Child
Gender	7 Mothers and 5 fathers	8 Girls and 4 boys
Mean age (range)	43 (31–63)	10 (1–19)
Age at first diagnosis		Prebirth to 10 years
Examples of diagnoses		ADHD Autism spectrum disorder Cerebral palsy Cystic fibrosis Hydrocephalus Intellectual disability Epilepsy Genetic syndrome Tracheostomy

Abbreviation: ADHD, attention deficit hyperactivity disorder.

### 2.3 Data collection

Data were collected using a proforma that consisted of three open‐ended, text‐box survey/interview questions (Box 1). This approach was the best option to collect rapid qualitative data before the interstate borders opened and not overburden parents. Proformas have been used successfully in other qualitative research^32^, ^33^ and are designed to encourage expansive answers from participants.^33^ Interviews were also offered. The questions were developed by S. S., E. M. (experienced qualitative researchers) and L. J. (an experienced parent representative). The questions were open‐ended to allow the participants to write or speak freely about their experiences. The questions were not piloted. For parents completing the questions by email, implicit informed consent was obtained when the participant completed and returned the form by email. Consent was provided verbally on the recording for parents who chose to be interviewed. Ten parents completed the open‐ended proforma, and two parents opted to complete the questions via an interview with a female qualitative researcher, S. S. Interviews were semi‐structured, audio recorded and transcribed verbatim. The two interviews were 26 and 44 min long. As expected, the amount of data was larger from the interviews. Yet, data were found to contain rich information in both the interviews and proformas allowing exploration into WA parents' stress at the beginning of COVID‐19.

### 2.4 Ethical considerations

Ethical approval was obtained from the Child and Adolescent Health Service Human Research Ethics Committee (RGSS0000003233), and reciprocal approval was obtained from two universities in Perth, Australia. Research Governance approval was obtained from the tertiary children's hospital in WA.

### 2.5 Data analysis

The data were analysed using Braun and Clarke's^34^, ^35^ reflexive thematic analysis. This is a method for identifying, analysing and reporting themes and patterns within data and is an appropriate approach for qualitatively exploring the life experiences of underrepresented groups.^36^ This study is positioned within the interpretivist paradigm, using a reflexive approach.^37^ Since the lived, subjective experiences of parents caring for children with long‐term conditions was an interest, and understanding the meanings that participants attributed to their stress experiences, and the subjectivity of the researchers' perspectives is acknowledged,^34^ reflexive thematic analysis was an appropriate methodology. Braun and Clarke's^34^, ^35^ six phases (familiarisation with the dataset, coding, generating initial themes, developing and reviewing themes, refining, defining and naming themes and writing up) were followed. S. S. and E. M. were involved in the analysis process. The two analysts each familiarised themselves with the responses independently. Data were read and reread and initial notes and thematic labels were recorded based on initial impressions. An inductive approach was followed with semantic (surface, obvious, overt) and latent (implicit, underlying, hidden) meanings generated from the data.^37^ NVivo^38^ was used to manage the data. Noticeable patterns were collaboratively discussed. Eight open‐ended proforma responses were doubled coded and the coding compared, and themes were refined and defined through regular meetings to advance interpretation. S. S. finalised the coding on the remaining four datasets, and any new insights were discussed between S. S. and E. M. Themes were continually reflected on and refined through this iterative process and through team discussions that involved returning to the proformas and interview transcripts. Themes were assigned that conveyed the shared meaning experienced by the participants. For example, the subtheme ‘hopes for a new normal’ was initially considered for a theme title but was revised to a subtheme as ‘COVID‐19 silver linings’ captured the theme. Thematic maps were used to collate codes and data items relative to the respective themes and aided to review the connections and implement revisions^37^ (see Supporting Information). All authors reviewed the final interpretations. Feedback assisted further engagement with the data and the final interpretations. The review and refinement also continued into write‐up.

## 3 FINDINGS

Two themes were produced. ‘Keep my child safe’ covers the parents' fears due to their children's health vulnerabilities, the adjustments made and the consequences experienced to protect their child. ‘COVID‐19's silver lining’ highlights the positives that resulted from the COVID‐19 pandemic, including their children having fewer infections, telehealth appointments provided, relationship improvements and the parent's hopes for a new normal. Key themes and related subthemes are outlined in Table 2.

**Table 2:** Key themes and related subthemes.

Theme 1: Keep my child safe
Subthemes
Vulnerable children
Adjustments to stay safe
Consequences
Theme 2: COVID‐19's silver linings
Subthemes
Fewer infections
Telehealth
Relationship improvements
Hopes for a ‘new normal’

Abbreviation: COVID‐19, coronavirus disease 2019.

### 3.1 Keep my child safe

#### 3.1.1 Vulnerable children

The parents in this study had children with long‐term conditions of which some, according to the parents, were more vulnerable to infections. Therefore, the parents were afraid and feared COVID, especially at the beginning of the pandemic, when little was known about the virus:

When Covid first hit, it was a very worrying time for our family, as we were very concerned for the health/life of our medically fragile child if he was to get COVID. (Sadie)

The parents became afraid of running out of medical supplies and medication their children depended on. Ruth highlighted the negative impact that could occur with missing just one dose of medicine for her son:

Because you've got to have medication…If he misses one dose, it increases his thing [condition] to happen more…So, if I can't get hold of his medicine…then…you're a bit dire straits [state of extreme distress].

Another fear was that their children would need hospital care for reasons other than COVID because they could not trust that the hospital was a safe place due to the pandemic. For example, one child had to attend the hospital monthly for blood transfusions. Parents mentioned that in the early stages, there was not enough hand sanitiser and masks and a lack of social distancing, as described by Emma:

Every day I'd walk in when my son was admitted and there would be piles of people standing in a group waiting to have their temperature checked and sign in online.

Similarly, there was frustration with others who did not follow the rules when attending hospital appointments which put their children at risk. Ruth described the seriousness of her child's condition and her anger at others dismissing the hospital rules:

I don't want to lose my child because somebody who I was sitting next to just can't be arsed…we'd enter into clinic and be sat out waiting with people that obviously had like common colds and stuff. But on the [hospital name] form, it said, do not enter if a cough, cold, or been in contact with someone with COVID.

The parents noticed that in the beginning, the staff at the hospital also expressed stress about the situation. According to the parents, the staff treated all children as infectious and demanded they wear masks. This behaviour made the parents feel that their children were contagious rather than vulnerable to catching the virus:

Drs and Nurses speak to him like he's a walking biohazard and not a patient. They don't consider their choice of words and can be quite offensive whether they meant to or not. (Emma)

The fear took other turns later during the pandemic, and the parents mentioned various worries, as outlined by Bronwyn:

The stress of caring for our child with complex needs was significantly increased…how we would manage in continuing to care for our daughter if either of us caught the virus but more importantly what it would mean if she caught the virus and the unknown outcome…a vaccine also presents its own concerns related to the risk of potential side effects as our daughter has previously had reactions to other forms of immunisation.

#### 3.1.2 Adjustments to stay safe

Many families were used to being isolated, keeping their vulnerable children away from crowds and minimising visitors due to their children's normal sensitivity to infections. For these families, COVID‐19 was not a huge difference. However, many parents like Brad stated that they had become even more ‘vigilant’ to try and continue to protect their children. For example, Emma described the precautions she took when shopping:

…it was a bit stressful in the early months just going to the supermarket and worrying that you could be taking coronavirus home on a box of [soda name]. I managed to get through this time though by wiping everything with [disinfectant name] wipes.

The adjustments parents made to feel safer during the pandemic included taking the children and their siblings out of daycare and school to minimise contact with other families. Some also stopped assistance with their child's physical and practical support with daily living:

Firstly, the need to stop all contact with our in‐home support workers to minimise our daughter's exposure to the risk of contracting COVID‐19. (Bronwyn)

Families undertook different daily activities due to the restrictions and social distancing that represented space and concealment:

…visit the ducks or play with a ball in the field during the pandemic. (Ella)

…we ended up going for some more drives really. (Brad)

Due to the closing of borders and isolation, some parents lost the usual support from grandparents or other extended family members who used to assist with the children's care and provide respite for the parents:

We relied heavily on family assistance before COVID and now have very little support from them. (Blake)

To handle home‐schooling and the children's daily care needs, some parents took leave from work to stay home and care for their children. Other parents could work from home. Parents who could do neither managed by taking turns and working in shifts. Doing so ensured that someone could always be at home with the child. Yet, one parent felt like they had become a single parent when their partner was kept away due to restrictions.

#### 3.1.3 Consequences

The consequences of the adjustments were that the parents became the sole carers of the children, who needed extensive help with daily living activities. In combination with isolation from family and friends, this increased anxiety and stress and decreased psychological well‐being and mental health for both parents and children:

This put quite a lot of stress on us as a family. (Sadie)

In all the effort to make the family stay safe, one parent described the impact on their daughter who had struggled with low mood, had dropped out from school and developed severe anxiety and self‐harming behaviour. Similarly, one father described how COVID‐19 resulted in him needing to take antidepressants as the additional stress he was now under was unbearable:

COVID's the final straw.

This highlights the additional burden that COVID‐19 brought to the already stressful environment of parental caregiving.

The new restrictions parents had to follow including one parent attending hospital appointments was challenging. Brad described his trials with needing his carer to attend the appointments, which allowed him to focus on the consultation:

I emailed ahead to say…I need to have an assistance with me…she [daughter] needs two people to hoist, she needs two people to change her etc…So, I got permission via the email. But still, when we went into clinics, I was met with abrupt, ‘one person only’. And I said, ‘No,’…‘I've already emailed her medical team. I've got permission for my carer to be here to help me’. And some people were ‘ok’, and some people just want to stress the point to the point we were at loggerheads.

Other consequences included delayed development and progress with some of the children's medical conditions. Moreover, parents worried about delayed or cancelled appointments with the healthcare. This concern was very much about problems that are not life‐threatening but important for the child's quality of life, for progress with speech and learning and for hindering regression. Delayed appointments resulted in delayed diagnoses and nonacute surgeries, thus impacting the child's daily life. Especially hard was not knowing for how long the delay should continue:

My son's [condition] appointments seem to have gone out the window. We were inpatients in July and we still have not been followed up [four months later]. (Annette)

Many parents were left wondering about the direction when the appointments were cancelled or delayed and received a lack of advice to help them care for their child during COVID‐19. Bronwyn highlighted the additional responsibility on parents when face‐to‐face appointments stopped:

…meant they were unable to directly observe or interact with our daughter and relied solely on us communicating all relevant information.

Likewise, the additional responsibility continued with the parents' new and unexpected role of home‐schooling their child, as Ruth described:

he had to be home schooled, which was a rude awakening for me. And I had to be up to date with where he was at school…Even though I'm a stay‐at‐home carer for him, learning all about what he's doing in school, progress and all that was even more intensive.

A few parents revealed that their children struggled with being out of routine with school and sports, and one struggled to get their child to wear a mask due to their sensory issues. When shops and supermarkets were left bare through panic buying, this caused difficulty for parents when they could not get specific products for their children:

there was no food at all really, it just went overnight…trying to get the toilet roll that he's used to because he only likes set ones, as you can imagine, no toilet rolls. (Ruth)

Couldn't get access to our regular hand gel and masks. (Hollie)

COVID‐19 impacted and heightened issues for some children, which caused added pressure on parents. Getting ill for some of the children could trigger their conditions, as Ruth outlines:

For him, it made him paranoid, which then I've got to keep a child having a meltdown next to me [at a hospital appointment] who thinks he's going to get sick, have a high fever.

When the closed borders were in place, and there was no transmission in the state, parents relaxed and let the children return to school and daycare. Due to the closed borders, many families felt safe. Being able to meet with family and friends again increased their well‐being. However, due to the closed border, some families were still left without the usual support from extended family living in other states.

Similarly, parents could not also visit ill relatives in other states and countries as they could not risk the possible barriers of not being able to return:

…my Mum died in [Country]…I could fly to [Country] for the funeral but I wouldn't get back. And if I could get back, I'd spend two weeks in quarantine away from [Child's Name] and the house, which is more added pressure on the wife and stuff, and funding for carers. (Brad)

When the interstate borders were planned to open, the threat of COVID‐19 came back, and the parents started to worry about their child's safety again.

### 3.2 Covid‐19's silver linings

#### 3.2.1 Fewer infections

Keeping the children out of daycare and minimising contact kept the children healthier due to fewer infections and colds. This was a positive side of COVID‐19 and something the parents mentioned as a welcoming change:

We did notice that while our child wasn't attending daycare it has been the best health we have ever seen him have. He didn't have any cold or flu symptoms, and no sickness. (Ella)

It also helped that more people washed their hands regularly and used hand sanitiser and masks, as outlined by Emma:

The positive impact was seeing the general public using hand gel as they walk into hospitals. We all hope that this becomes the norm, as it should be.

Parents felt it was okay to now talk about hand hygiene and encourage and remind guests to use hand sanitiser before entering their home, which had been a difficult conversation before.

#### 3.2.2 Telehealth

One of the good things about isolation and physical distance was that the children could have more telehealth appointments (telephone and virtual) instead of needing to go to the hospital for a face‐to‐face meeting. This saved a lot of time, especially for families living outside the metropolitan area. It also saved money and energy when they did not have to travel:

The main change is the reduction of travel as we are now able to access telehealth and we have been able to negotiate more [treatment] via [online platform] or similar, freeing valuable time and reducing stress. (Elliot)

Telehealth did not suit all kinds of appointments, which was disappointing for some parents:

We attempted to link in with [various therapy] sessions using online meeting platforms, but this was not very useful for our daughter and her condition which is generally benefitted from face‐to‐face sessions to retain attention and maximise learning. (Luke)

Yet, despite this, parents were pleased that the sessions were available. Some of the services did not offer virtual appointments which at times was hard for parents to receive a satisfying experience in their child's care. Brad described the difficulties he experienced and the need for all modes of telehealth to be available for parents:

My daughter…was meant to have a follow‐up for her dental surgery she had, and they wanted to do the dental over the phone. And I said, well, ‘how are you going to see?’ you know, cause obviously it's a follow‐up to having teeth removed and gums cut into to remove teeth…I said how you gonna see anything wrong over the phone, when you're not even doing a video call?

The parents also emphasised the importance of using equipment that the parents could access from home instead of equipment that had to be accessed at the local healthcare clinic. The necessity to go to a healthcare facility instead of accessing telehealth from home hindered them from keeping physical distance:

They want us to attend a Community Centre [town name] for our next video conference which is a place where a lot of small kids and babies attend. (Emma)

#### 3.2.3 Relationship improvements

While COVID‐19 was a worrying time; it also provided time to review life and a bridge for relationship barriers. One of the positive outcomes of COVID‐19 was that families had more time together:

…we stopped to appreciate the small things and enjoyed quality time together. (Sadie)

For Ruth, the restrictions had made her change general practitioners (GPs) as she wanted to attend a GP service that was implementing social distancing for her son's safety. This resulted in receiving unexpected additional support for her child and improved care that she had not experienced before:

…more of a relationship with the new GP. I found out through him that my son didn't have [an additional condition], which was a good thing because I was told by the old GP that he did. But the new GP was more proactive with getting him referrals to [hospital].

Many parents praised the various professionals that had assisted them in general, from healthcare professionals who provided reassurance for their child, pharmacists ensuring medications were available and schoolteachers ensuring their child was kept up to date.

For the first time, the parents expressed a relief that others could now have a better understanding of their lives:

Awareness. Our smaller and broader community understands the isolation and fear that WE LIVE during Flu season. (Hollie)

#### 3.2.4 Hopes for a ‘new normal’

The parents reflected on several recommendations to continue to keep their children safe. It was suggested that the hand sanitiser needs to be available at various locations rather than just in the clinics:

I was quite disappointed when I posted on [hospital opinion website] asking that we could have hand gel at the entrances to [hospital] and still even during COVID this wasn't done. (Emma)

There should also be more hand sanitiser stations at [hospital] around car parks, lifts, etc. (Sadie)

To reduce exposure to COVID, parents recommended that there be one entrance and one exit at hospitals and safety measures to continue:

We would like to see a continuation of many of the safety/security measures (social distancing, hand health, numbers, screening etc) continue on into the future. (Elliot)

Similarly, better safety measures at the hospital were mentioned by several parents:

During the pandemic there needed to be better policing of public places such as throughout [hospital]. Throughout the pandemic, measures set up to monitor and control the public's access to various areas of the hospital were inconsistent and often ad hoc. (Bronwyn)

A few parents mentioned having advice available to reduce panic and having an advocate. Byron hoped that the previous experiences with COVID‐19 may help in the future:

Everyone was making policy on the run. If ever the situation arises again, I'm sure things will be better communicated and executed.

Parents also wanted the possibility of telehealth to be a sustainable option:

Clearly for us the continuation of telehealth and [online platform] [condition] [treatment] would be a great assistance. (Elliot)

Online home schooling and new technologies for teaching opened new possibilities. The parents could see opportunities with these new ways of teaching and how they could continue to use them in the future. For instance, the children may not need to stay out of school the whole day because of a hospital appointment in the middle of the day. Or they could do home‐schooling from the hospital bed:

We now have systems in place for keeping up with school and perhaps attending appointments with minimal disruption to [daughter's name] learning journey. (Hollie)

Parents also suggested similar interventions that were set up for the elderly with special shopping times to also be done for parents with vulnerable children. Some parents did not have the support available and had to take their child shopping with them, risking exposing them to the virus. Similarly, it was recommended to streamline hospital appointments to prevent multiple visits to reduce exposure.

## 4 DISCUSSION

The findings from this exploratory qualitative study reveal insights into how the initial stages of COVID‐19 impacted parents caring for children with long‐term conditions in WA. Parents experienced more stress during the start of COVID‐19 when much was unknown about the virus. It was revealed that the main course of action for the parents was to keep their child safe which resulted in various adjustments (e.g., keeping their child home from school) and experienced various consequences from the pandemic (e.g., further isolation and access to healthcare services). Yet, positives were also noted during the start of COVID‐19, including their children having fewer infections, some appointments being via telehealth which helped with the often difficult task of getting their child to hospital appointments, and relationship improvements within healthcare (professionals and services) and more broadly. For some families, there was the hope that these improvements would continue and become the ‘new normal’.

Understanding parents' stress responses is essential, especially when caring for vulnerable children where experiences of stress are likely to be enhanced due to COVID‐19.^39^ Stress in parents caring for vulnerable children during the COVID‐19 pandemic has been related to diagnosis, prescribed medication of the immunosuppressed child, geographical location, household composition and employment status of the parent.^39^ In the current study, additional stresses were found to be caring for their child while home‐schooling, working from home and having increased parenting demands (e.g., absence of partners who could not return to WA due to the border restrictions and/or support services stopped).

The ‘tend and befriend’ theory^40^ is an interesting approach applicable to the current study's context. It focuses on children and states that when faced with a perceived threat, people tend to their young and rely on others for connection and support. It was initially stated that females tended to their children and sought social connection,^40^ whereas males were more likely to follow the fight‐or‐flight response.^41^ There is much debate about gender and stress when caring for children with long‐term conditions.^13^, ^42^ The current study did not aim to study gender differences. However, participating fathers and mothers both tended to their children by attempting to keep them safe, as noted previously.^13^

The theory implies that stress levels may decrease when social interactions are comforting.^43^ It is well known that parents caring for children with long‐term conditions can experience a lack of support from family, friends and healthcare services.^13^, ^44^ Social isolation was a common theme on the impact of COVID‐19 with family caregivers of individuals with end‐stage heart failure and lifestyle changes were noted in the United States study.^45^ The parents in the current study were limited in befriending others due to COVID‐19 consequences, which caused further feelings of isolation. Despite many parents feeling that border restrictions were needed to protect their children, the impact of this was that families were alienated from their key social support which they relied upon. This theory aids to highlight the greater level of stress parents experienced. To keep their children safe, they were unable to access vital social support via friends and family. Yet, a few experienced unexpected support from healthcare professionals (e.g., obtaining medications) and professionals (e.g., teachers help in home schooling) who assisted them during stressful times. These positive experiences were helpful to parents and beneficial in moving forward at this time of crisis.

While the parents described that others now had a better understanding of their daily lives, the fear of their child getting COVID persists. The parents provided many recommendations, including separate entrances and exits at the hospital to avoid unnecessary queues, having hand sanitisers and masks available and staff monitoring who was entering. Procedures and staff were unprepared at the start of the pandemic, and these recommendations have since been implemented at the children's hospital. More than ever during COVID times, additional support is required to access healthcare online or at the hospital to ease the burden for these parents.^13^, ^14^ Parents also need to protect themselves to prevent passing COVID to their children and to be able to care for their children, especially when support may be limited. Similarly, the implications of long COVID need to be considered. Further research into parents' COVID experiences and long COVID is necessary to explore parents' stress experiences when caring for children with long‐term conditions to assist this group.

Telehealth was a benefit for some of the parents and something they would like to continue. Telehealth assisted with prompt appointments and reduced the travel and difficulties most encounter when taking their child to the hospital. It also enabled parents and their children to keep safe from COVID. In support, telehealth has been found to improve the provision of health services and be a critical tool.^46^ However, the current study highlights that not all appointments were suitable for telehealth. Previous research has also noted reduced hospital admissions for children with long‐term conditions in paediatric wards.^47^ The current study highlights that suitable adjustments need to be in place for parents caring for children with long‐term conditions (e.g., appropriate telehealth appointments and allowing a support person to assist a parent at hospital appointments) for future outbreaks and pandemics.

The current study suggested that some children had become distressed with the threat of COVID and the changes to their routines. Child and family distress have been noted to be heightened due to the messages about the use of handwashing, sanitisers, mask use, social distancing and so forth.^48^ Children and young people's experiences in WA and internationally have been explored during COVID‐19 through open‐ended surveys and drawings.^48^, ^49^ An international study exploring the experiences of lockdown through children's artwork created an ebook reflecting children's experiences during COVID‐19.^48^ It was recommended that healthcare professionals need to support the child's health literacy, make them feel secure and take into consideration their hopes, fears and worries.^48^ The authors suggest that the ebook may assist with starting conversations with children about the impact of COVID‐19. Communication is key for keeping children informed and to promote wellbeing, but how best to do this for children with long‐term conditions requires further investigation.

Each family in the current study is unique with personal backgrounds/histories and stressors. Still, their collective experiences at the start of the COVID‐19 pandemic and support needs are alike. This is in agreement with previous research from WA, where families of children with medical complexity describe their support needs similarly despite the complexities.^13^, ^14^

## 5 LIMITATIONS AND STRENGTHS

Participants were recruited from one family support organisation and English speaking, which may limit the applicability of the findings. No single parents were involved in the study; this group is expected to experience higher stress levels. Limitations can be attributed to the proforma via email, as further probing was not possible. However, the interview format was offered, and two parents chose this option. The study was conducted during the COVID‐19 pandemic and captured the stressful experiences at a particular time in WA that was unique to other states and countries due to the border restrictions.

## 6 CONCLUSION

The COVID‐19 pandemic increased stress on parents of children with long‐term conditions, who were already psychosocially vulnerable. Policy makers, researchers, government and community services need to consider how to safely adjust restrictions and provide support to enable these families to better cope during a pandemic. Key areas include promoting safe access to their child's healthcare requirements and their social networks. COVID‐19 also led to some welcome changes and outcomes for these families. Further research is needed to better understand how these ‘silver linings’ can be harnessed after COVID‐19.

## AUTHOR CONTRIBUTIONS

Stephanie Smith, Evalotte Mörelius made substantial contributions to the conception and design of the study, conducted the qualitative analysis and were involved in the interpretation of the data and writing of the findings. Stephanie Smith conducted and transcribed the interviews and drafted the manuscript. Mary Tallon, James Smith and Lauren Jones consulted on the study. Stephanie Smith, Mary Tallon, James Smith, Lauren Jones and Evalotte Mörelius critically reviewed the manuscript for important intellectual content and gave final approval of the version to be published.

## CONFLICT OF INTEREST STATEMENT

The authors declare no conflict of interest.

## Supporting information

Supporting information.

## Data Availability

The datasets presented in this article are not readily available because the data are based on sensitive information from parents' caring for children with long‐term conditions. Requests to access the datasets should be directed to the corresponding author.

## References

[1] World Health Organisation . *Rolling updates on coronavirus disease (COVID‐19)*. 2020. Accessed February 19, 2022. https://www.who.int/emergencies/diseases/novel-coronavirus-2019/events-as-they-happen

[2] Hacker KA , Briss PA , Richardson L , Wright J , Petersen R . COVID‐19 and chronic disease: the impact now and in the future. Prev Chronic Dis. 2021;18:210086. 10.5888/pcd18.210086 PMC8220960 34138696

[3] Mörelius E , Munns A , Smith S , et al. Pediatric and child health nursing: a three‐phase research priority setting study in Western Australia. J Pediatr Nurs. 2022;63:39‐45. 34973465 10.1016/j.pedn.2021.12.016

[4] Commissioner for Children and Young People Western Australia . *Profile of children and young people in WA 2021*. 2021. Accessed February 17, 2023. https://www.ccyp.wa.gov.au/media/4578/profile-of-children-and-young-people-in-wa-2021-report-final-web-version-february-2021.pdf

[5] Australian Bureau of Statistics . *Snapshot of Western Australia—high level summary data for Western Australia in 2021*. 2022. Accessed February 11, 2023. https://www.abs.gov.au/articles/snapshot-wa-2021

[6] Thomas HM , Runions KC , Lester L , et al. Western Australian adolescent emotional wellbeing during the COVID‐19 pandemic in 2020. Child Adolesc Psychiatry Ment Health. 2022;16(1):4. 35027061 10.1186/s13034-021-00433-y PMC8756750

[7] Government of Western Australia . *WA confirms first novel coronavirus death*. 2020. Accessed August 11, 2021. https://ww2.health.wa.gov.au/Media-releases/2020/WA-confirms-first-novel-Coronavirus-death

[8] Nathan A , George P , Ng M , et al. Impact of COVID‐19 restrictions on Western Australian children's physical activity and screen time. Int J Environ Res Public Health. 2021;18(5):2583. 33807520 10.3390/ijerph18052583 PMC7967372

[9] Bhoyroo R , Chivers P , Millar L , et al. Life in a time of COVID: a mixed method study of the changes in lifestyle, mental and psychosocial health during and after lockdown in Western Australians. BMC Public Health. 2021;21(1):1947. 34702238 10.1186/s12889-021-11971-7 PMC8547299

[10] Australian Government Department of Health . *The Australian Health Sector emergency response plan for novel coronavirus (short form)*. 2020. Accessed May 24, 2022. https://www.health.gov.au/resources/publications/australian-health-sector-emergency-response-plan-for-novel-coronavirus-covid-19-short-form

[11] Rose J , Willner P , Cooper V , et al. The effect on and experience of families with a member who has intellectual and developmental disabilities of the COVID‐19 pandemic in the UK: developing an investigation. Int J Dev Disabil. 2020;1‐3. 10.1080/20473869.2020.1764257 PMC8928853 35309704

[12] Spinelli M , Lionetti F , Pastore M , Fasolo M . Parents' stress and children's psychological problems in families facing the COVID‐19 outbreak in Italy. Front Psychol. 2020;11:1713. 32719646 10.3389/fpsyg.2020.01713 PMC7350926

[13] Smith S , Tallon M , Clark C , Jones L , Mörelius E . “You never exhale fully because you're not sure what's NEXT”: parents' experiences of stress caring for children with chronic conditions. Front Pediatr. 2022;10:902655. 35832577 10.3389/fped.2022.902655 PMC9271768

[14] Moyes A , Abbott T , Baker S , Reid C , Thorne R , Mörelius E . A parent first: exploring the support needs of parents caring for a child with medical complexity in Australia. J Pediatr Nurs. 2022;67:e48‐e57. 36192287 10.1016/j.pedn.2022.09.018

[15] Masa'Deh R . Perceived stress in parents of children with chronic disease: a comparative study. Eur Sci J. 2015;36:389‐400.

[16] Australian Institute of Health and Welfare (AIHW) . *Australia's children*. 2020. Accessed February 17, 2023. https://www.aihw.gov.au/getmedia/6af928d6-692e-4449-b915-cf2ca946982f/aihw-cws-69-print-report.pdf.aspx?inline=true

[17] Geweniger A , Barth M , Haddad AD , et al. Impact of the COVID‐19 pandemic on mental health outcomes of healthy children, children with special health care needs and their caregivers–results of a cross‐sectional study. Front Pediatr. 2022;10:759066. 35223688 10.3389/fped.2022.759066 PMC8866820

[18] Houtrow A , Harris D , Molinero A , Levin‐Decanini T , Robichaud C . Children with disabilities in the United States and the COVID‐19 pandemic. J Pediatr Rehabil Med. 2020;13(3):415‐424. 33185616 10.3233/PRM-200769

[19] Crawley E , Loades M , Feder G , Logan S , Redwood S , Macleod J . Wider collateral damage to children in the UK because of the social distancing measures designed to reduce the impact of COVID‐19 in adults. BMJ Paediatr Open. 2020;4(1):e000701. 10.1136/bmjpo-2020-000701 PMC7223269 32420459

[20] Mielke J , Leppla L , Valenta S , et al. Unraveling implementation context: the Basel Approach for coNtextual ANAlysis (BANANA) in implementation science and its application in the SMILe project. Implement Sci Commun. 2022;3(1):102. 36183141 10.1186/s43058-022-00354-7 PMC9526967

[21] Myers‐Walls JA . Family life education for families facing acute stress: best practices and recommendations. Fam Relat. 2020;69(3):662‐676.

[22] Martinsone B , Tzivian L . Differences in stress and coping during the COVID‐19 pandemic in families with and without children with developmental disorders or chronic conditions. Front Public Health. 2021;9:704577. 34490186 10.3389/fpubh.2021.704577 PMC8417897

[23] Horesh D , Brown AD . Traumatic stress in the age of COVID‐19: a call to close critical gaps and adapt to new realities. Psychol Trauma. 2020;12(4):331‐335. 32271070 10.1037/tra0000592

[24] Fava GA , McEwen BS , Guidi J , Gostoli S , Offidani E , Sonino N . Clinical characterization of allostatic overload. Psychoneuroendocrinology. 2019;108:94‐101. 31252304 10.1016/j.psyneuen.2019.05.028

[25] Leigh‐Hunt N , Bagguley D , Bash K , et al. An overview of systematic reviews on the public health consequences of social isolation and loneliness. Public Health. 2017;152:157‐171. 28915435 10.1016/j.puhe.2017.07.035

[26] Western Australian Government . *Controlled interstate border*. 2020. Accessed February 19, 2022. https://www.wa.gov.au/government/announcements/controlled-interstate-border

[27] Conradi L , Hazen A , Covert J . The fast and the furious: the rapid implementation of tele‐mental health practices within a Children's Advocacy Center. Glob Implement Res Appl. 2022;2:305‐320. 36348723 10.1007/s43477-022-00065-0 PMC9633018

[28] Smith J , Rapport F , O'Brien TA , et al. The rise of rapid implementation: a worked example of solving an existing problem with a new method by combining concept analysis with a systematic integrative review. BMC Health Serv Res. 2020;20(1):449. 32438909 10.1186/s12913-020-05289-0 PMC7240003

[29] Quanbeck A , Hennessy RG , Park L . Applying concepts from “rapid” and “agile” implementation to advance implementation research. Implement Sci Commun. 2022;3(1):118. 36335373 10.1186/s43058-022-00366-3 PMC9636827

[30] Slattery P , Saeri AK , Bragge P . Research co‐design in health: a rapid overview of reviews. Health Res Policy Syst. 2020;18(1):17. 32046728 10.1186/s12961-020-0528-9 PMC7014755

[31] Smith S , Mörelius E . *Collaborating with parents of children with long‐term conditions: research Australia*. 2021. Accessed March 6, 2022. https://issuu.com/researchaustralia/docs/inspire_issue_21/42

[32] Smith J , Braithwaite J , O'Brien TA , et al. The voices of stakeholders involved in precision medicine: the co‐design and evaluation of qualitative indicators of intervention acceptability, fidelity and context in PRecISion Medicine for children with cancer in Australia. Qual Health Res. 2022;32:1865‐1880. 36066496 10.1177/10497323221120501

[33] Smith J , Lee MD , Ellis LA , Ijaz K , Yin K . Developing a novel psychographic‐behavioral qualitative mapping method for exergames. Int J Serious Games. 2021;8(2):87‐107.

[34] Braun V , Clarke V . Reflecting on reflexive thematic analysis. Qual Res Sport Exerc Health. 2019;11(4):589‐597.

[35] Braun V , Clarke V . Using thematic analysis in psychology. Qual Res Psycho. 2006;3(2):77‐101.

[36] Casey LJ , Bowman SJ , Power E , McAloon J , Wootton BM . A cognitive‐behavioral exploration of the psychological impact of the Australian Marriage Law postal survey: a reflexive thematic analysis. Psychol Sex Orientat Gend Divers. 2023;10(1):91‐102.

[37] Braun V , Clarke V . One size fits all? What counts as quality practice in (reflexive) thematic analysis? Qual Res Psychol. 2021;18(3):328‐352.

[38] QSR International Pty Ltd . NVivo qualitiatve data analysis software. 12th ed., 2018.

[39] Driessens C , Mills L , Culliford D , et al. Parental concern for clinically vulnerable child during first 18 months of the COVID pandemic. Pediatr Res. 2022;1‐9. 10.1038/s41390-022-02371-7 PMC9684768 36418484

[40] Taylor SE , Klein LC , Lewis BP , Gruenewald TL , Gurung RAR , Updegraff JA . Biobehavioral responses to stress in females: tend‐and‐befriend, not fight‐or‐flight. Psychol Rev. 2000;107(3):411‐429. 10941275 10.1037/0033-295x.107.3.411

[41] Cannon WB . The wisdom of the body. Norton; 1932.

[42] Cousino MK , Hazen RA . Parenting stress among caregivers of children with chronic illness: a systematic review. J Pediatr Psychol. 2013;38(8):809‐828. 23843630 10.1093/jpepsy/jst049

[43] Taylor SE . Tend and befriend: biobehavioral bases of affiliation under stress. Current Direct Psychol Sci. 2006;15(6):273‐277.

[44] Smith J , Cheater F , Bekker H . Parents' experiences of living with a child with a long‐term condition: a rapid structured review of the literature. Health Expect. 2015;18(4):452‐474. 23311692 10.1111/hex.12040 PMC5060798

[45] Cross LA , Koren A , Dowling JS , Gonzales JE . The impact of COVID‐19 on family caregivers of ndividuals with end‐stage heart failure. J Hosp Palliat Nurs. 2022;24(5):249‐257. 35881680 10.1097/NJH.0000000000000881 PMC9435954

[46] Monaghesh E , Hajizadeh A . The role of telehealth during COVID‐19 outbreak: a systematic review based on current evidence. BMC Public Health. 2020;20(1):1193. 32738884 10.1186/s12889-020-09301-4 PMC7395209

[47] Gavish R , Levinsky Y , Dizitzer Y , et al. The COVID‐19 pandemic dramatically reduced admissions of children with and without chronic conditions to general paediatric wards. Acta Paediatr. 2021;110(7):2212‐2217. 33539565 10.1111/apa.15792 PMC8013809

[48] Foster M , Al‐Motlaq M , Carter B , et al. Seeing lockdown through the eyes of children from around the world: reflecting on a children's artwork project. Nurs Praxis Aotearoa N Z. 2021;37:104‐115.

[49] Commissioner for Children and Young People Western Australia . *COVID‐19: as told by WA children and young people*. 2020. Accessed February 17, 2023. https://www.ccyp.wa.gov.au/media/4350/covid-19-as-told-by-wa-children-and-young-people-july-2020.pdf

