# Supplementary material for: COVID‐19 in Western Australia: ‘The last straw’ and hopes for a ‘new normal’ for parents of children with long‐term conditions
Source: Health Expect. 2023 Jun 12;26(5):1863–73. doi: 10.1111/hex.13792 (PMC10485346; doi:10.1111/hex.13792)

**Supplementary Information: Thematic Maps**

**COVID-19 in Western Australia: *“The last straw”* and hopes for a *“new normal”* for parents of children with long-term conditions.**

Smith, S., Tallon, M., Smith, J., Jones, L., Mörelius, E. (2023)


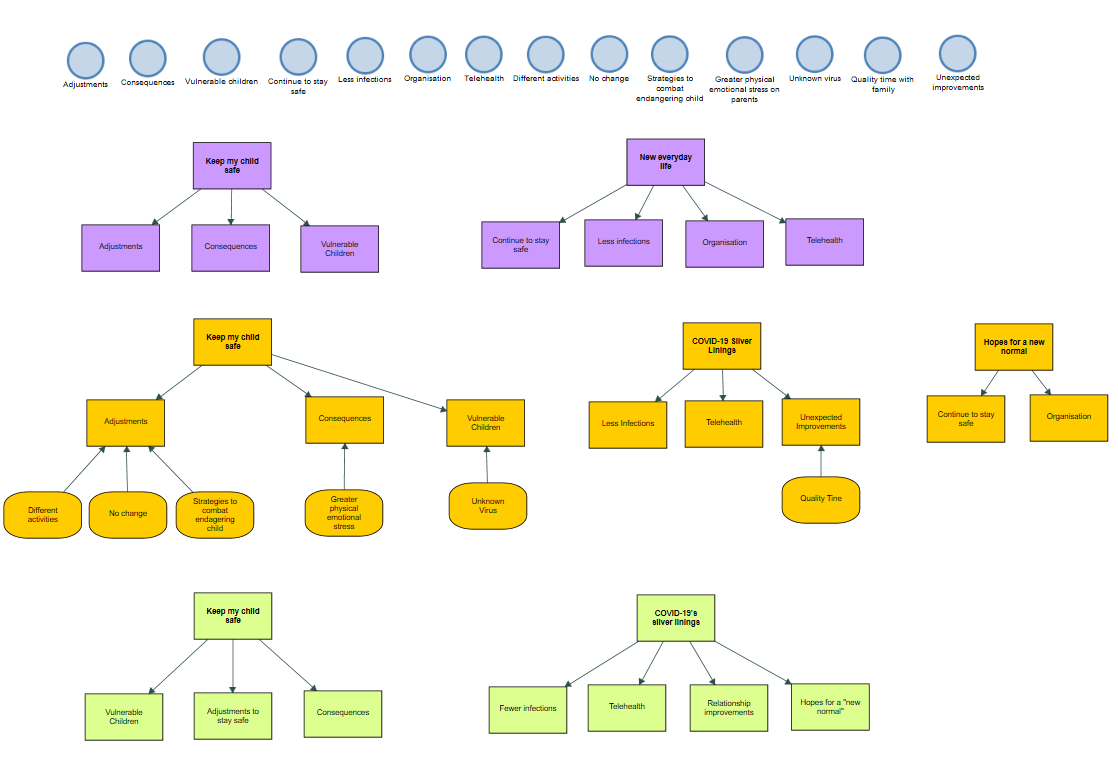

Supplement: Supplementary file 1 — Supporting information. [file HEX-26--s001.docx]
